# Comment on Tremmel et al. In Vitro Metabolism of Six C-Glycosidic Flavonoids from *Passiflora incarnata* L. *Int. J. Mol. Sci.* 2021, *22*, 6566

**DOI:** 10.3390/ijms23084445

**Published:** 2022-04-18

**Authors:** Monika Beszterda, Rafał Frański

**Affiliations:** 1 Department of Food Biochemistry and Analysis, Poznań University of Life Sciences, Mazowiecka 48, 60-623 Poznań, Poland; monika.beszterda@gmail.com; 2 Faculty of Chemistry Adam Mickiewicz University, Uniwersytetu Poznańskiego 8, 61-614 Poznań, Poland

In recent years, growing attention has been paid to the chemical composition of aerial parts extracts and the bioavailability of active compounds from different species of *Passiflora* genus [1,2,3]. Detailed information on the absorption and metabolism of dietary flavonoids in the digestive tract are important for determining their physiological functions. The main constituents of passionflower are O- and C-glycosidic flavonoids and secondary metabolites, representing up to 2.5% of the dried plant; however, β-carboline alkaloids, essential oils, and carbohydrates have also been reported. Compared with O-, N- and S-glycosides, C-glycosides are quite stable against chemical and enzymatic treatments [4], and deglycosylation is a crucial reaction for the absorption and/or exertion of the biological activities of various chemicals [5]. Until now, in vitro and in vivo studies of the metabolism of C-glycosidic flavonoids have been quite insufficient.

In the study of Tremmel et al., an attempt was made to characterize the metabolism of C-glycosidic flavonoids with luteolin or apigenin basic structures linked to one or two sugar residues, using the Caco-2 monolayer cell line as the in vitro metabolism model [6]. Caco-2 cells mimic, both physically and biochemically, the human intestinal epithelial membrane, including passive diffusion as well as active and passive transport. This permeability model permits both apical to basolateral and basolateral to apical transport to be explored, with the apical compartment mimicking the intestinal lumen and the basal compartment mimicking the bloodstream [7,8,9]. Tremmel et al. examined the distribution of the potential metabolites, and samples were collected from the cell lysate, as well as from the apical and basolateral compartments [6]. As described in detail, the enzymes expressed by the Caco-2 model potentially cleave the C-C glycosidic bond in vitro, which has not been confirmed thus far. Among the tested C-glycosides, orientin, isoorientin and isovitexin exhibited a broader metabolism in phases I and II, where hydroxylated and methoxylated products could be detected, whereas sulfates dominated as conjugation substrates. In contrast, vitexin, schaftoside and isoschaftoside showed a narrow diversity of the metabolite profiles and lacked the presence of sulfate and glucuronic acid as phase II products. Moreover, the authors suggested that the preferred sulfation of flavonoids could be explained by *ortho*-dihydroxy groups at C-3′/C-4′ in the B rings of the selected flavonoids. Most of the identified products of the metabolism in vitro were detected in the apical compartment, which simulates the intestine. In this space, these metabolites act as substrates of the gut microbiota, allowing them to form further metabolites.

In summary, this study offers important knowledge regarding the in vitro metabolism of C-glycosidic flavonoids from *Passiflora incarnata* L. (C-glycosidic flavones can be efficiently metabolized in the intestine) [6]. Because they could play a significant role as bioactive compounds, considering their accumulation in medicinal plants (i.e., *Passiflora*, *Crataegus*, *Viola*) and their pharmacological activity, further structure–metabolism relationships and research on enteroenteric and enterohepatic circulations are required.

An issue, which in our opinion was not sufficiently described by Tremmel et al., was the identification of metabolites by using UHPLC-MS/MS [6]. The identification of compounds using a MS/MS experiment must be based on the detection of respective product ions. The only product ions reported by the authors were those presented in Table 1. In our opinion, the detection of most of those ions could not be expected for the metabolites presented in Table 1, as explained below.

1. Quercetin glucuronide: Two product ions were reported, at *m/z* 301 and *m/z* 176. The first was formed by the loss of glucuronide moiety (loss of mass 176) and it is typical fragmentation of flavonoid glucuronides [10,11,12,13]. However, the second product ion at *m/z* 176 cannot be formed from deprotonated quercetin glucuronide. The ions at *m/z* 176 would be the odd-electron ones corresponding to the glucuronide moiety (in this case, radical anions) and these types of product ions are not observed upon the fragmentation of flavonoid glycosides/glucuronides in either positive or negative ion mode;
2. Luteolin glucuronide: Product ions were not detected by the authors; the simple question is why?
3. Methoxy luteolin: At first it should be mentioned that the [M-H]^−^ ion at *m/z* 299 corresponds to the O-methyl luteolin (not methoxy luteolin). The authors claim the detection of the product ion at *m/z* 285; thus, the loss of mass 14 (elimination of CH_2_ moiety). However, the characteristic feature of the fragmentation of [M-H]^−^ ions of methoxylated flavonoids (flavone, flavonol, or isoflavone) is the loss of mass 15 (loss of methyl radical) [14,15,16,17]. For diosmetin (4′-O-methyl luteolin), the elimination of the CH_2_ moiety was proposed [18], in our opinion, not correctly. It is worth adding that none of the mass spectra of methoxylated flavonoids deposited in the spectral database show the product ions formed by the loss of CH_2_ moiety from [M-H]^−^ ions (https://massbank.eu/MassBank/, accessed on 11 January 2022);
4. Luteolin sulfate: The product ion at *m/z* 285, and thus the loss of mass 80 (elimination of the SO_3_ molecule), is a typical fragmentation of flavonoid sulfates [13,19]. Therefore, the detection of this metabolite is plausible;
5. Methoxy luteolin sulfate: Analogically as above, it should be O-methyl luteolin sulfate. The formation of the product ion at *m/z* 299 is reasonable (loss of mass 80), but the formation of the product ion at *m/z* 285 cannot be expected;
6. Orientin/isoorientin sulfate: The detection of the product ion at *m/z* 327 (the loss of mass 200) is reasonable. This product ion can be formed by the loss of mass 120 (typical fragmentation of C-glycosides [16,20,21,22]) and by the loss of mass 80 (typical fragmentation of flavonoid sulfates [13,19]). However, the authors claim that the product ion at *m/z* 327 was formed by the loss of C_6_H_12_O_6_ and SO_3_. The loss of C_6_H_12_O_6_ would correspond to the loss of the hexose molecule, which for C-glycosides is impossible. Furthermore, the loss of C_6_H_12_O_6_ would correspond to a loss of mass 180; thus, the product ion would have a different *m/z* value. For the isoorientin sulfate, the authors claim the detection of product ion at *m/z* 80 (odd-electron ion [SO_3_]^−.^). The formation of such an ion, under the condition used, cannot be expected;
7. Methoxy-isoorientin sulfate: The detection of the product ion at *m/z* 461 is reasonable (although formally it should be O-methyl isoorientin sulfate).

From the text in the manuscript, it can be expected that the MS/MS data, and thus the respective product ions, are included in the Supplementary Materials, e.g., “The sulfated orientin and isoorientin monoconjugates could be confirmed through HPLC-DAD-MS/MS analysis (Tables S7–S14)…”. However, in the Supplementary Materials, the product ions are not included, and the tables contain only the data of accurate mass measurements. Although the obtained accurate mass measurements confirm the detection of the proposed metabolites, the addition of the observed product ions is strongly desirable. We suggest the authors show at least some of the obtained MS/MS spectra (for the most abundant metabolites), maybe as a reply to our comment or as corrigendum to their paper, since it surely would improve the paper. 

It has to be stressed that there is no doubt that the paper by Tremmel et al. [6] is of a very high scientific level, and our very specific comments concerning the product ions do not affect this.

## Data Availability

Not applicable.
